# Meta-analysis reveals evolution in invasive plant species but little support for Evolution of Increased Competitive Ability (EICA)

**DOI:** 10.1002/ece3.488

**Published:** 2013-02-14

**Authors:** Emmi Felker-Quinn, Jennifer A Schweitzer, Joseph K Bailey

**Affiliations:** Department of Ecology and Evolutionary Biology, University of Tennessee – Knoxville569 Dabney Hall, Knoxville, Tennessee, 37996, USA

**Keywords:** Defense tradeoffs, evolution of increased competitive ability (EICA), herbivory, introduced range, invasive plant species, plant defense, rapid evolution, selective agents

## Abstract

Ecological explanations for the success and persistence of invasive species vastly outnumber evolutionary hypotheses, yet evolution is a fundamental process in the success of any species. The Evolution of Increased Competitive Ability (EICA) hypothesis (Blossey and Nötzold 1995) proposes that evolutionary change in response to release from coevolved herbivores is responsible for the success of many invasive plant species. Studies that evaluate this hypothesis have used different approaches to test whether invasive populations allocate fewer resources to defense and more to growth and competitive ability than do source populations, with mixed results. We conducted a meta-analysis of experimental tests of evolutionary change in the context of EICA. In contrast to previous reviews, there was no support across invasive species for EICA's predictions regarding defense or competitive ability, although invasive populations were more productive than conspecific native populations under noncompetitive conditions. We found broad support for genetically based changes in defense and competitive plant traits after introduction into new ranges, but not in the manner suggested by EICA. This review suggests that evolution occurs as a result of plant introduction and population expansion in invasive plant species, and may contribute to the invasiveness and persistence of some introduced species.

## Introduction

In the research effort to identify and explain the success of invasive species in their new range, evolutionary explanations for invasiveness are rarely invoked. Only a small proportion of introduced species succeed in their new range, some attaining greater individual size or fitness or establishing populations of greater numbers or densities, than what might be predicted from their native range (Hinz and Schwarzlaender 2004; but see Thebaud and Simberloff 2001). Bossdorf et al. (2005) divided research into invasiveness as seeking either an ecological or an evolutionary explanation and determined that research efforts have been heavily skewed toward identifying ecological explanations. More recently, 26 of the 29 hypotheses of plant invasion identified in a recent review (Catford et al. 2009) explain invasions as the result of static plant traits, suitability of the invaded environment, or ecological interactions between species traits and environments. Studies that link invasions to evolutionary interactions between invasive plants and their new environment represent a small fraction of invasive species research (Lee 2002; Kollmann and Banuelos 2004; Prentis et al. 2008; Alexander et al. 2009; Allan and Pannell 2009; Hornoy et al. 2011; Haider et al. 2012), despite the importance of local adaptation in determining the distribution and success of some native plant species (Macel et al. 2007; Alvarez et al. 2009; Kawakami et al. 2011). The most prominent of the hypotheses of plant invasion which invoke evolution of invasives, the Evolution of Increased Competitive Ability (EICA) hypothesis (Blossey and Nötzold 1995), proposes that evolution of invasive populations occurs as a release from coevolved herbivores, not in response to selective factors in the environment of the new range.

Evolution of Increased Competitive Ability identifies herbivores as the primary selective agent for shifts in defense and growth characteristics in invasive populations. Blossey and Nötzold (1995) suggested that the species *Lythrum salicaria* allocates significant resources to defenses against coevolved specialist herbivores in its native range, and this allocation constrains growth of the species, as suggested by optimal defense theory. In the invasive range of *L. salicaria* where the coevolved herbivore is absent, plant allocation shifts from defenses to higher growth, and these reduced defenses and greater allocation to growth become genetically fixed in invasive populations. Herbivore release has been experimentally confirmed as a selective agent in *Oenothera biennis*, where herbivore exclusion by insecticide use leads to reductions in population frequencies of quantitative defense compounds within a few generations (Agrawal et al. 2012). However, few invasive species enjoy total release from herbivory (Müller-Schärer et al. 2004), particularly those species in which coevolved herbivores from the native range have been introduced either accidentally (Zangerl and Berenbaum 2005) or as part of classical biological control methods (Thomas and Reid 2007; Van Driesche et al. 2010). The Shifting Defense Hypothesis (SDH), which suggests that specialist-targeted defenses (digestibility reducers) will decrease as generalist-targeted defenses (leaf toxins) increase in invasive populations, was proposed as a refinement of EICA to better reflect the reality of partial release from herbivores in the invasive range (Müller-Schärer et al. 2004; Joshi and Vrieling 2005; Doorduin and Vrieling 2011). Herbivore release, however, may not be the only or even the primary selective pressure on invasive populations. A review by Colautti et al. (2009) of EICA studies suggested that shifts in performance traits for plant species in invasive ranges exhibit latitudinal clines, which may indicate adaptation to climate (Buswell et al. 2011). Stochastic, nonselective evolutionary processes (founder events, multiple introductions, hybridization, bottlenecks, isolation by distance; reviewed in Keller and Taylor 2008; see also Durka et al. 2005; Vasemagi 2006) also have the potential to alter defense and growth traits important to plant success. Studies designed to test the specific predictions of evolutionary change laid out by EICA may be used to evaluate evidence of evolutionary change in invasive plants without reference to putative selective or stochastic agents.

In order to provide a broad quantitative review, we tested the predictions of the EICA hypothesis for changes in plant defense and competitive ability using meta-analytic techniques. Published tests of EICA rarely quantitate the same specific plant traits, or use the same methods to evaluate defense or competitive ability: for example, defenses are evaluated by assessing leaf concentrations of quantitative or qualitative chemical defenses (e.g., secondary compounds), or by measuring the growth and fitness of herbivores feeding on the plants, or by quantitating the degree of damage to the plants themselves. This variety of response variables may explain why published reviews explicitly addressing EICA have been qualitative, assessing evidence by comparing the number of significant studies for or against the hypothesis (Hinz and Schwarzlaender 2004; Bossdorf et al. 2005). The limitations of vote-counting approaches, however, are that qualitative studies judge evidence based on the number of significant studies, without evaluating the magnitude of trait changes in those studies, and do not include nonsignificant results in assessing total evidence for changes in ranges. Similarly, a recent meta-analysis of plant defenses evaluating evidence for SDH (Doorduin and Vrieling 2011) does not address the central aspect of EICA, which is that reduced defenses should co-occur with greater plant vigor or fitness, that is, higher competitive ability, in invasive populations. For the purpose of this review, we grouped different experimental approaches to quantitating defense or plant competitive ability as testing separate predictions of the EICA hypothesis. For defense, EICA predicts that (1) when released from coevolved enemies in the home-range, introduced-range plants will evolve lower defenses; (2) When both the introduced- and home-range plants are subjected to feeding by a single species of herbivore, herbivores will feed with more success (fewer negative effects on development and survival) on introduced-range plants, leading to (3) higher rates of herbivory damage on plants from the introduced range compared with plants from the home range. In terms of competitive ability, EICA predicts that as defenses decrease, genetically based shifts in allocation will result in (4) higher growth, as well as (5) higher reproduction and fitness, leading to higher (6) competitive ability in introduced-range plants. Here, we use meta-analytic techniques to assess the evidence from published studies for each of these predictions. In addition to using meta-analysis to evaluate the EICA hypothesis, we used data from EICA studies to evaluate whether there are genetically based differences between home and introduced ranges in general across invasive plant species. Our results suggest that while there may be limited evidence for evolution of reduced defense and greater plant performance traits consistent with EICA, evidence for any evolution (selective or stochastic) of traits concurrent with introduction is stronger. Such evidence of genetic change in concert with plant invasion mandates greater attention in invasion research to the importance of stochastic and selective forces in the introduced range of plant species (*sensu* Buswell et al. 2011).

## Methods

In order to test the EICA hypothesis that there are genetically based differences between defense and growth traits of introduced- versus home-range populations of invasive plants, we collected published studies from peer-reviewed journals. For the purposes of this review, we are interested in modern invasions, not in range expansions or in historical introduction events. “Invasion” refers to the presence of a plant species novel to an area that was transported and introduced accidentally or intentionally by humans. We define “home” range as the continent where a species has been present since at least the time of European colonial expansion (c. 1500), and “introduced” range as a continent or group of continents where a species was not present preceding European colonialism.

We used the search terms “EICA” or “evolution of increased competitive ability” and “ecology” with lemmatization to collect 45 papers from Web of Science in December 2010. In order to be included in the analyses, papers had to meet each of the following criteria: (1) Papers presented data from experiments that tested at least one prediction of the EICA hypothesis using at least one invasive plant species. Thus, studies that presented only the results of models, or in one case applied the EICA model to a fish system, were excluded; (2) Plants from both the introduced and home ranges of each invasive species were grown in a common environment (greenhouse or common garden) so that any variation expressed was due to underlying genetic differences, not to differences in environment or plasticity in response to environment; (3) Each of the introduced and the home ranges were represented by at least two geographically distinct populations. EICA emphasizes the difference between ranges, each of which is comprised of many populations growing under different abiotic and biotic conditions specific to geographically distinct locations. A comparison of only two populations, one from each range, confounds local, population-specific genetic structure with the genetic constraints (stochastic and selective) specific to each range. Including at least two populations from each range ensures that the question of genetic differences between groups is addressed at the scale of range and not population. Following application of these criteria, 27 studies were included in the analysis from which we collected 347 observations of the difference between home and introduced ranges of invasive species in defense, growth, or competitive characteristics (Table 1).

**Table 1:** Sources of data used in meta-analysis. Sources are listed by year of publication, from earliest to latest

Publication	Species^1^	Leaf Traits^2^	Herbivore Response^3^	Plant Response^4^	Performance^5^	Competition
Willis et al. 1999;	*Lythrum salicaria*	M(C)	S(BR), G(BR)		V	
van Kleunen and Schmid 2003;	*Solidago canadensis*			D	V, R	
Blair and Wolfe 2004;	*Silene latifolia*	P			V, R	
Bossdorf et al. 2004;	*Alliaria petiolata*				V, R	X
Maron et al. 2004a,b;	*Hypericum perforatum*	M(W)		D		
Buschmann et al. 2005;	*Barbarea vulgaris*			G(NR), U(NR)	V, R	
Buschmann et al. 2005;	*Bunias orientalis*			G(NR), U(NR)	V, R	
Buschmann et al. 2005;	*Lepidium draba* (*Cardaria draba*)		G(NR)	G(NR), U(NR)		
Buschmann et al. 2005;	*Rorippa austriaca*			G(NR), U(NR)		
Cipollini et al. 2005;	*Alliaria petiolata*	M(C,I)				
Joshi and Vrieling 2005;	*Jacobaea vulgaris* (*Senecio jacobaea*)	M(C)	G(NR), S(NR)	G(NR)	V, R	
Meyer et al. 2005;	*Solidago gigantea*		U(NR)	U(NR), D	V,R	
Muller and Martens 2005;	*Lepidium draba* (*Cardaria draba*)	M(C)			V	
Stastny et al. 2005;	*Jacobaea vulgaris* (*Senecio jacobaea*)			S(NR)	V, R	
Guesewell et al. 2006;	*Solidago gigantea*				V, R	
Hull-Sanders et al. 2007;	*Solidago gigantea*	M(C,I)	G(BR), S(NR)			
Johnson et al. 2007;	*Solidago gigantea*	M(C)			V	
McKenney et al. 2007;	*Lepidium draba* (*Cardaria draba*)				V	X
Bossdorf et al. 2008;	*Senecio inaequidens*			G(IR)	V, R	
Eigenbrode et al. 2008;	*Cynoglossum officinale*	M(C,I)				
Franks et al. 2008;	*Melaleuca quinquenervia*	M(W), P	S(BR)		V	
Handley et al. 2008;	*Senecio vulgaris*				V, R	
Ridenour et al. 2008;	*Centaurea stoebe* ssp*. micranthos* (*Centaurea maculosa*)	M(C), P	G(BR), S(BR), G(NR)	G(BR), S(BR)	V, R	X
van Kleunen and Fischer 2008;	*Mimulus guttatus*				V, R	
Williams et al. 2008;	*Cynoglossum officinale*				V, R	
Zou et al. 2008;	*Triadica sebifera* (*Sapium sebiferum*)			U(NR)	V	X
Abhilasha and Joshi 2009;	*Conyza canadensis*		G(IR), S(NR)	G(IR)	V, R	
Cripps et al. 2009;	*Lepidium draba* (*Cardaria draba*)		U(BR)	S(NR)	V	
He et al. 2009;	*Centaurea stoebe* ssp*. micranthos* (*Centaurea maculosa*)				V	X
Huang et al. 2010	*Triadica sebifera* (*Sapium sebiferum*)	M(C)	G(NR), S(NR)	G(NR), S(NR)	V	

1 Species names verified by Integrated Taxonomic Information System. Names in parentheses indicate taxonomic synonyms used in EICA literature.

2 Leaf-level plant defensive traits in the form of secondary metabolites (M) or physical traits (P). Secondary metabolites were evaluated when expressed constitutively (C) or after induction by herbivory (I), or were measured without classifying herbivory (W).

3 Plant defenses were assessed via the effect of herbivory upon the survival, growth, or development of feeding insects. Herbivore species are specialists (S), generalists (G), or were observed as unclassified communities (U). The herbivore species were limited in their distribution to the invasive range of the plant species (IR), the native range of the plant species (NR), or was distributed across both invasive and native ranges of the plant species (BR).

4 Plant defenses assessed as the extent of herbivory, or the impact of herbivory on the survival, growth, or reproduction of plants. Herbivore species are specialists (S), generalists (G), or were observed as unclassified communities (U). In some studies, plant responses were to damages (D) caused by bacterial or fungal pathogens, or by herbivory simulated by clipping; these studies were excluded from categorical analyses shown in Figure 3b. The herbivore species were limited in their distribution to the invasive range of the plant species (IR), the native range of the plant species (NR), or was distributed across both invasive and native ranges of the plant species (BR).

5 Plant competitive ability assessed by performance of plants grown in common environments. Metrics of plant performance include measures of vegetative growth (V) and measures of reproductive effort (R).

Papers reported comparisons between introduced and home ranges as F-statistics, Chi-squared scores, and t-scores from statistical tests, and in a few cases as mean values and standard deviations for each range. Each observation was converted to a Fischer's Z transformation of the correlation coefficient, except for observations of competitive ability. Competitive ability results were analyzed as natural logarithm-transformed response ratios, as most studies reported comparisons of competitive abilities of home- and introduced-range plants in this form. Positive Z-scores (or response ratios) indicate that the value of the response variable is higher in the introduced range than the home range, and negative Z-scores indicate that the response value is higher in the home range than the introduced range. In the case of response metrics that relate to plant defense, all Z-scores were multiplied by an appropriate weighting variable (−1 or 1) so that negative scores represented higher inferred defenses in the home-range plants and positive scores represented higher inferred defenses in the introduced-range plants.

We characterized comparisons between introduced and home-range responses as either defense or competitive traits. There were three models that addressed components of the defense hypothesis. The first defense model included quantitative and qualitative leaf traits, such as concentrations of secondary compounds, density of trichomes, and leaf toughness. The second defense model included the effects of herbivory in home- versus introduced-range plants upon herbivore performance, and included metrics from choice experiments or garden surveys such as developmental time of insects, insect mass, and number of insects. The third defense model included herbivory-induced damage upon plants using metrics such as mass of plant consumed, area of leaves consumed, and regrowth following herbivory. All effect sizes were modeled randomly, which is appropriate for ecological studies in which variation in measured effects is comprised of biotic variation as well as error. In the case of significant summary effect sizes, fail-safe numbers (N_R_) were calculated to indicate the number of nonsignificant, unpublished results that would render the summary effect size nonsignificant. If N_R_ exceeded Rosenthal's identified minimum value (5n+10), the result was assumed to be robust against publication bias (Rosenthal 1979). We used Metawin 2 (Rosenberg et al. 2011) for all analyses.

In addition to assessing defense traits, we created models that addressed three components of the hypothesis that there is a difference between ranges in competitive ability. The first model included measures of plant performance related to growth, including height, biomass, and growth rate. These measures were taken from plants from introduced and home ranges when all are grown under noncompetitive conditions, either alone in pots or in common gardens. The second model included measures of plant investment in reproduction, that is, fitness, including floral and seed mass and number, and number of vegetative offspring in plants for which asexual reproduction is important. Although EICA as originally formulated did not make specific predictions for reproductive allocation, reproductive traits have been correlated to abundance of invasive plant species within communities (Lloret et al. 2005). The third model included results from direct tests of the relative competitive ability of home- versus introduced-range plants. Competitive ability was measured by growing target plants with intraspecific competitors, interspecific competitor plants from the introduced range, or interspecific competitor plants from the home range. Only results in which the target plant of competition was the invasive species were included, so the test for competitive ability was of the invasive species' relative ability to withstand competition from another plant. Results in which the target plant was another species from the community, which would measure the impact of the invasive species upon other species, were excluded from this analysis. Since more recently published EICA studies tended to use refinements of earlier experimental design and more appropriate nested statistical models, we also ran models using year of publication as an explanatory variable for each of the defense and growth traits. Year of publication did not significantly explain variation in any of the defense or growth effect sizes (*P* > 0.4), indicating that improvements in experimental or analytic techniques were unlikely to explain trends in data.

We also addressed the hypothesis that there were genetically based differences or evolutionary change between ranges in defense or competitive ability, regardless of whether it was consistent with EICA. We ran random effects models of the three types of defense characteristics and three types of characteristics that address plant performance and competitive ability, models in which all effect sizes were positive. This allows evaluation of whether any evolutionary change has occurred concurrent with invasion and establishment of a new range across all invasive plant species. In this case, any effect size with a confidence interval that does not overlap zero indicates that there is a significant difference between home and introduced ranges in a quantitative trait, without indicating broad trends in direction of trait change.

## Results

### Defense Characteristics in the EICA framework

**Figure 1:**
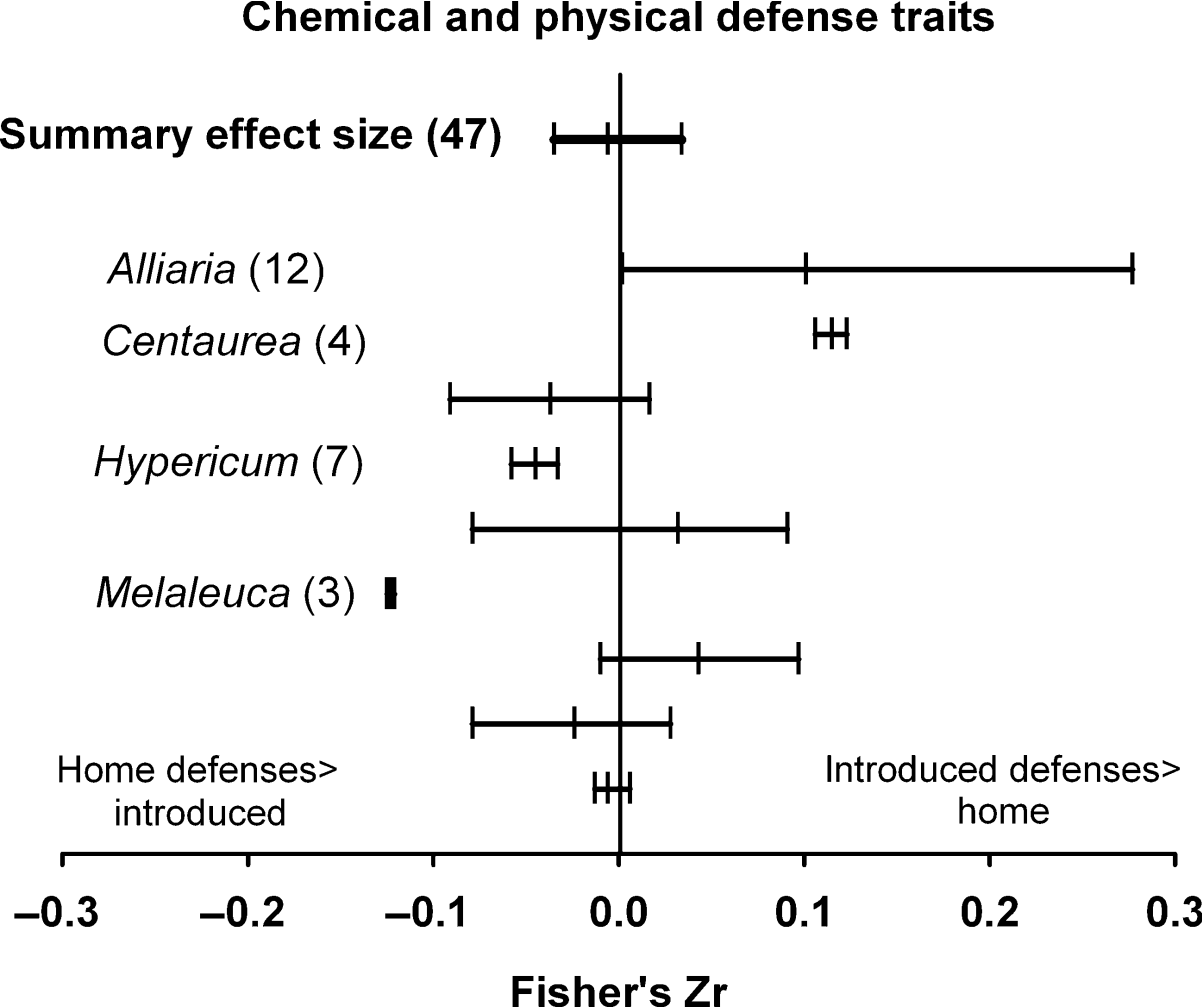
Chemical and physical defense traits in introduced- versus home-range invasive plant species. The summary effect which includes all contrasts is at the top of the graph. Effects are grouped by plant species, and species in which there was a significant effect are indicated by genus name on the graph. Numbers in parentheses indicate the number of contrasts of introduced- versus home-range plants summarized by each effect. Error bars indicate bias-corrected 95% confidence intervals, and error bars that overlap the *y*-axis indicate an effect which is not statistically significant (i.e., there is no significant difference between home and introduced ranges for this effect).

**Figure 2:**
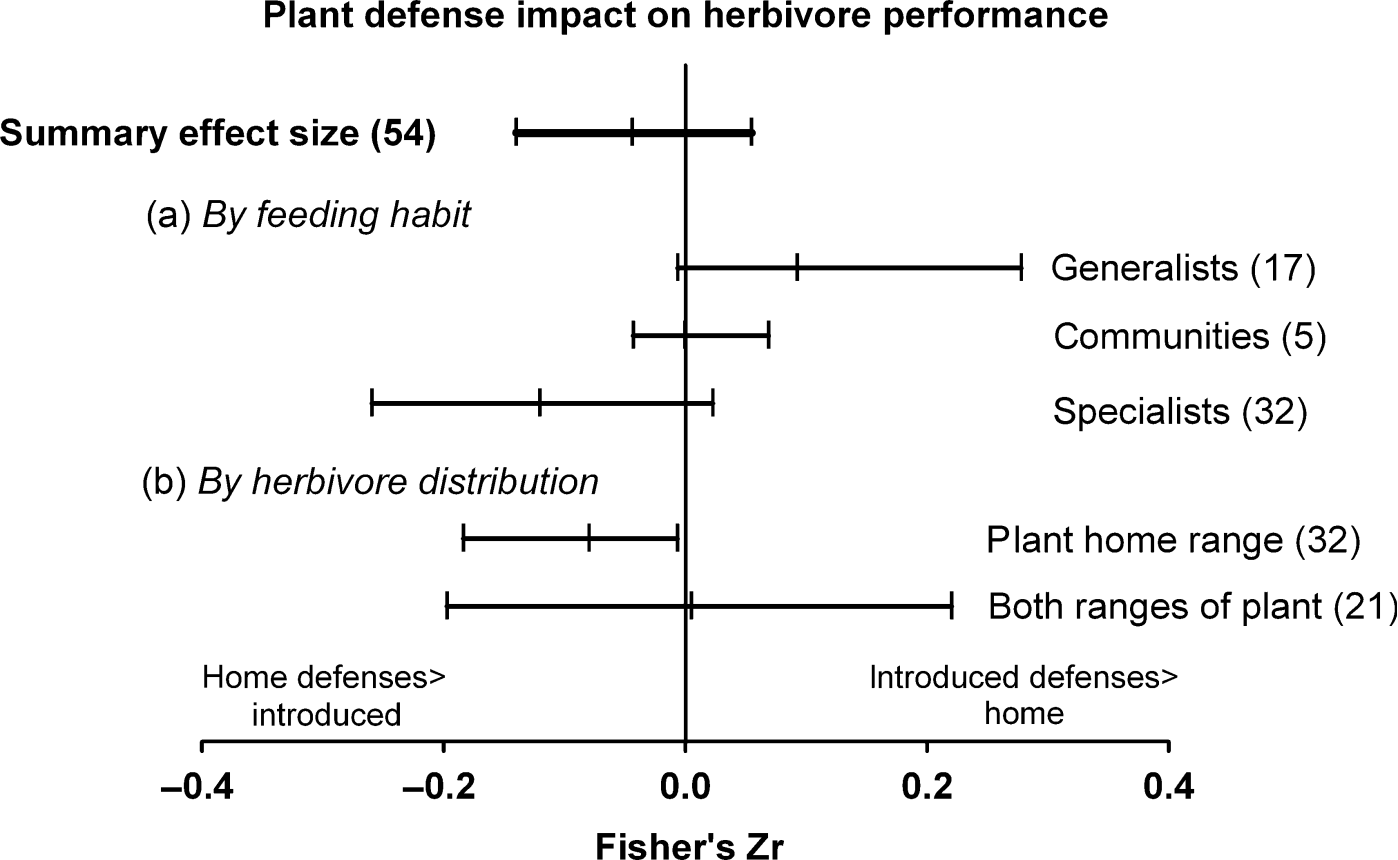
Plant defenses evaluated via herbivore performance, herbivores fed on introduced- versus home-range plants. The summary effect which includes all contrasts is at the top of the graph. Effects are categorized by a) herbivore feeding habit or degree of specialization, or by b) herbivore distribution in the range of the plant species evaluated. Numbers in parentheses indicate the number of contrasts of introduced- versus home-range plants summarized by each effect. Error bars indicate bias-corrected 95% confidence intervals, and error bars that overlap the *y*-axis indicate an effect which is not statistically significant (i.e., there is no significant difference between home and introduced ranges for this effect).

**Figure 3:**
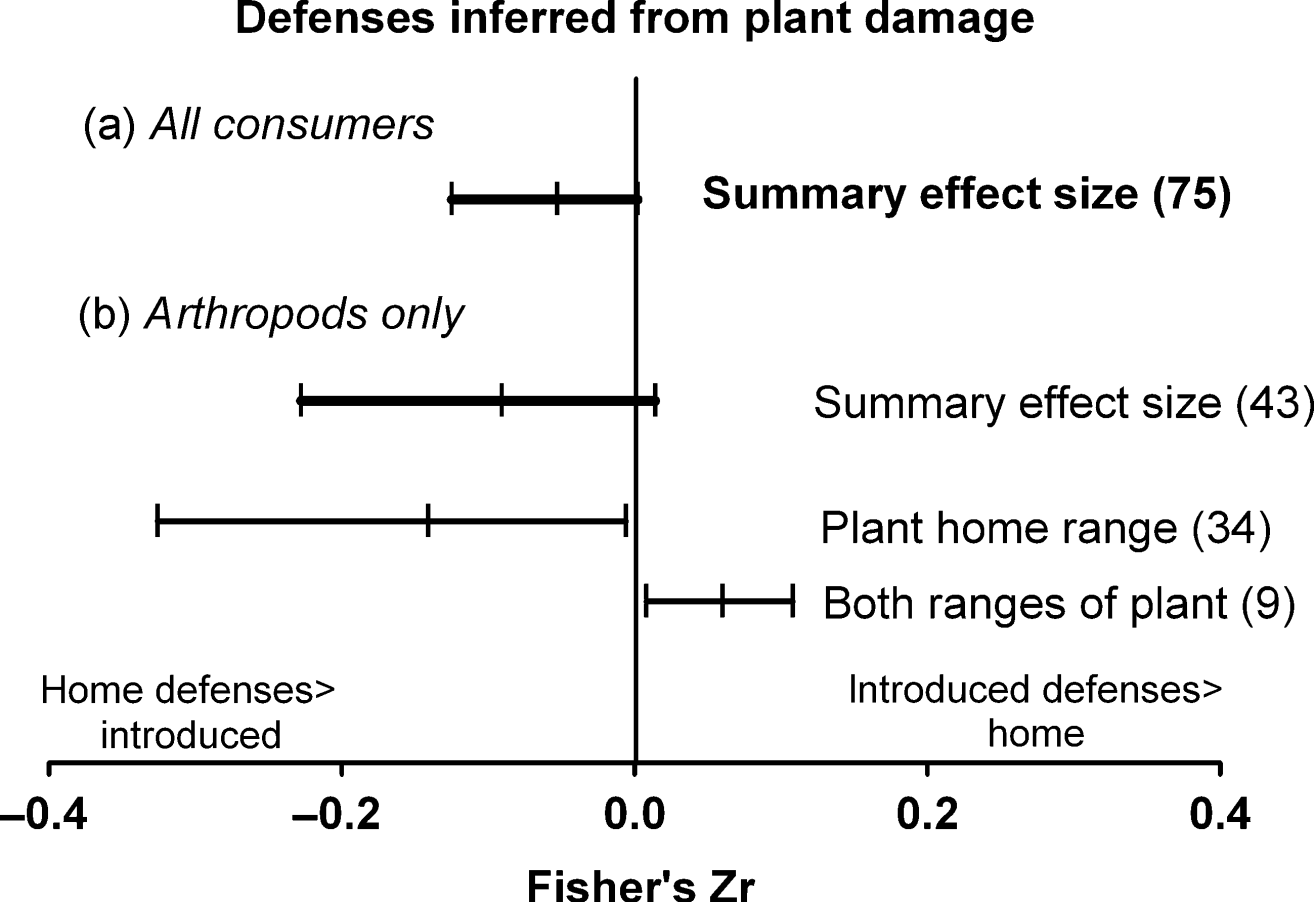
Plant defenses inferred from plant damage by herbivores, in introduced- versus home-range plants. The a) overall summary effect which includes all contrasts is at the top of the graph. Plant damage caused by b) arthropod herbivores (plant pathogens and mechanical damage excluded) is shown in summary effect and also categorized by herbivore distribution in the ranges of the plant. Numbers in parentheses indicate the number of contrasts between home and introduced ranges of invasive plant species for each class of trait. Error bars indicate bias-corrected 95% confidence intervals, and error bars that overlap the *y*-axis indicate an effect which is not statistically significant (i.e., there is no significant difference between home and introduced ranges for this effect).

We found no general support across invasive plant species for reduced defenses in the introduced range of invaders. There were no overall differences between home and introduced ranges within each species in defense characteristics measured as leaf chemical and physical traits (Fig. 1), their effect on herbivore performance (Fig. 2), or relative herbivore damage to plants (Fig. 3). However, heterogeneity indices indicated that variance in each model could be explained by factors other than geographic range. Chemical and physical leaf defenses varied significantly by plant species, which explained 66% of the variation in effect sizes (Fig. 1; Q = 23.6765, *P* = 0.002), indicating that there are a few species which support the defense predictions of EICA. We also considered whether expression of defenses would explain variation in leaf chemistry effect sizes, but found no difference in overall effect size between constitutive and induced defenses (Q=4.2512, *P* = 0.236).

Although there were no significant differences overall by plant range for herbivore performance or plant damage inflicted by herbivores, further classification of herbivores revealed significant variation in effect sizes for these metrics. Herbivore family did not explain variation in herbivore performance (*P* = 0.86) or plant damage by herbivores (*P* = 0.76), but classifying herbivores by degree of feeding specialization or geographic range explains some of the heterogeneity in effect sizes. Classifying herbivores by feeding habits—generalist, specialist, or unclassified communities of herbivores—predicted 67% of the variation in effect size of herbivore performance, although these categories were not statistically significant (Fig. 2, *P* = 0.048). There were trends toward a decline in performance of generalists and a rise in performance of specialists when both were placed on introduced plants, indicating that introduced plants tended toward higher defenses against generalists and lower defenses against specialists than home-range plants. However, the degree of herbivore specialization did not significantly explain variation in defenses as inferred from the amount of herbivore damage sustained by plants (*P* = 0.64). Herbivores were also categorized by geographic range; herbivores collected from the plant species' home range were more negatively impacted by feeding on home-range plants, while herbivores present in both ranges due to universal distribution or human introduction as biocontrol agents were equally impacted by defenses from home- versus introduced-range plants (Fig. 2). Herbivore geographic range also explained variation in plant damage by herbivores: home-range plants suffered less damage from herbivores restricted to the home range, indicating higher defenses in the home range against accustomed predators, while introduced-range plants suffered less herbivore damage from herbivores currently found in both ranges, indicating greater defenses in introduced-range plants against universally distributed and human-introduced herbivores (Fig. 3). Plant species did not significantly predict variation in effect sizes in difference by range for herbivore performance (*P* = 0.098) or herbivore-induced damage to plants (*P* = 0.067).

### Performance and competitive ability in the EICA framework

**Figure 4:**
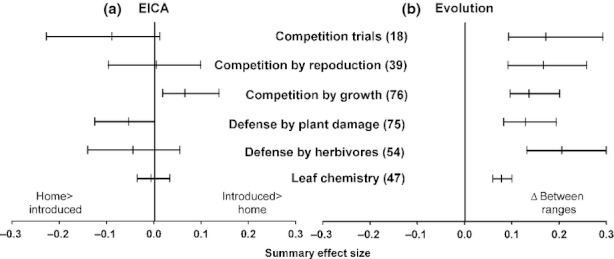
Meta-analysis of plant defense and competitive traits showed no broad support for a) EICA hypothesis, but general support for b) evolution of all traits in the introduced range of invasive plant species. Panel (a) shows all EICA summary effect sizes, and panel (b) shows all summary effect sizes evaluating the hypothesis that evolution occurs with invasion. Numbers in parentheses indicate the number of contrasts between home and introduced ranges of invasive plant species for each class of trait. Note that all effect sizes are Fisher's Z-transformations, except for the competition trial effect sizes, which are natural logarithm-transformed response ratios. Error bars indicate bias-corrected 95% confidence intervals, and error bars that overlap the *y*-axis indicate an effect which is not statistically significant (i.e., there is no significant difference between home and introduced ranges for this effect).

**Figure 5:**
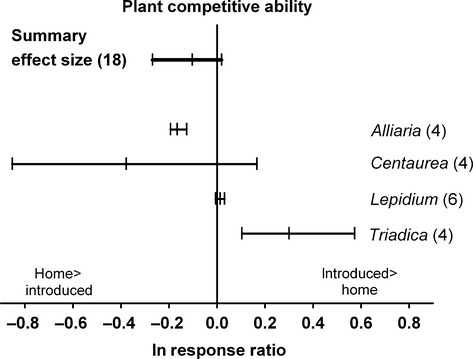
Plant competitive ability in introduced versus home ranges of invasive plant species. The summary effect which includes all contrasts is at the top of the graph. Effects categorized by species are indicated by genus names. Numbers in parentheses indicate the number of contrasts between home and introduced ranges of invasive plant species for each class of trait. Error bars indicate bias-corrected 95% confidence intervals, and error bars that overlap the *y*-axis indicate an effect which is not statistically significant (i.e., there is no significant difference between home and introduced ranges for this effect).

There was mixed support for EICAs prediction that introduced-range plants would have higher competitive ability than their home-range relatives within each species. Grown in a common, low- or no-competition environment, introduced-range plants had significantly higher measures of nonreproductive performance and vigor than did home-range plants (Fig. 4), with 48% of the variation in effect size explained by species (*P* = 0.0154). The fail-safe number for this result suggests that this effect is robust against publication biases. However, there was no corresponding difference by range in plant fitness (Fig. 4) or in plant performance under competitive conditions (Fig. 5). Plant species did not significantly explain variation in fitness (*P* = 0.811), although it did explain variation in competitive ability (*P* = 0.02, Fig. 5). The low number of studies (five studies containing 18 results) that published the results of experiments that evaluated the response of invasive populations to competition means that this result should be interpreted with caution.

### Defense and competitive ability in evolutionary framework

All defense and competitive traits varied significantly by range when direction of response was disregarded in order to address the hypothesis that plant traits evolved in response to introduction and expansion in a new geographic range (Fig. 4). Chemical and physical leaf defense traits varied significantly by range, as did herbivore performance on plants from different ranges and herbivore-induced plant damage. Plant performance under noncompetitive conditions varied significantly by range, as did plant fitness and plant competitive ability. Fail-safe numbers indicate that each of these effects is unlikely to be an artifact of publication bias.

## Discussion

This meta-analysis shows that there is little general support for the specific predictions of the EICA hypothesis across published tests of the hypothesis, but broad support for evolutionary change co-occurring with the introduction and invasion of plant species. EICA predicts that there will be reduced defenses against herbivores in the introduced range, but effect summaries do not indicate widespread reduction in a range of defense traits in introduced ranges as categorized by leaf's physical and chemical traits, effects on herbivores, and herbivore damage to plants. EICA also predicts that there will be increased plant performance and competitive ability in the introduced range, and while there was higher performance in the introduced range of invasive plants, it was under noncompetitive garden conditions which may not mimic species interactions that occur in nature. Fitness traits did not increase in the introduced range across the invasive species considered, and the few direct measures of competition did not indicate a general increase in competitive ability. Although we did not find broad support for EICA, each of the defense and competitive characteristics demonstrated significant divergence between home and introduced ranges across all the invasive species considered. While this meta-analysis shows that herbivore release does not generally act as a selective force on plant allocation between defense and growth, it does show that stochastic or selective forces are broadly important and that evolutionary divergence occurs between introduced-range plants and parental-range plants in the course of plant invasion.

### Defense Traits and EICA

Contrary to the EICA hypothesis, there was no reduction in defense in the introduced ranges of invasive species across the 19 studies (176 comparisons) in which some metric of defense was evaluated, which suggests that release from herbivory is not in general a powerful selective force upon plant defenses in invasive species. We found no support for EICA's prediction that leaf chemical and physical defense traits will in general be reduced in the introduced range. A recent meta-analysis which evaluated putatively generalist-specific defenses did find support for higher levels of leaf toxins in introduced ranges, as suggested by the SDH (Doorduin and Vrieling 2011). This difference in result is due to differences in selection criteria for papers and data; Doorduin and Vrieling (2011) were interested in evaluating the more specific SDH, and used 13 measures of leaf toxins from nine studies, whereas our meta-analysis of leaf chemistry and physical traits used 39 measures of secondary metabolites along with eight measures of physical defenses from 12 studies. We included all reported tests of secondary chemistry (e.g., both induced and constitutive levels of defenses) in the meta-analysis, as we were testing the broader EICA hypothesis, and recent work suggests that selection criteria should be tested as explanatory factors in meta-analysis rather than being used to exclude data (Lajeunesse 2010), which may bias results toward supporting prominent theories (Barto and Rillig 2012). Our inclusive datasets of chemical and physical leaf traits, herbivore performance, and herbivore-induced plant damage allowed us to evaluate proposed refinements of EICAs defense predictions. For example, release from herbivory has been hypothesized to differentially affect the evolutionary trajectory of secondary chemicals based on their expression, leading to reduction in constitutive defenses and a compensatory increase in induced defenses (Koricheva et al. 2004). However, we found no differences by ranges in effect sizes based on the expression of putative defenses (constitutive vs. induced). With regard to the SDH hypothesis (Müller-Schärer et al. 2004; Joshi and Vrieling 2005; Doorduin and Vrieling 2011), herbivore performance in our analysis suggests a trend toward differences in response between specialists and generalists congruent with SDH, but the confidence intervals associated with each effect (Fig. 2) show that published evidence is insufficient to support a difference between introduced- and home-range plant defenses against either class of herbivores across invasive plant species. Moreover, these trends did not lead to specialists or generalists causing greater plant damage based on plant range. The most powerful explanatory factor of the degree of herbivore-induced plant damage was the geographic range of the herbivore. Home-range plants were more heavily defended than introduced-range plants against herbivores restricted to the plant's native range, which would appear to support EICA. However, introduced-range plants were more heavily defended, suffering less herbivore attack and damage, than home-range plants against widely distributed herbivores, including specialist herbivores that had been introduced to the range as biocontrol agents. This suggests that introducing insect species to invasive populations may result in the evolution of invasive plants more resistant to or tolerant of herbivory, as has been documented with invasive *Pastinaca sativa* following the accidental introduction of its herbivore *Depressaria pastinacella* to North America (Zangerl and Berenbaum 2005). This analysis suggests that the efficacy of biological control agents should be evaluated based on ranges where plants are well-defended as well as ranges where lower defenses have evolved. This paper shows that the EICA hypothesis' predictions about defenses are not broadly supported across invasive species.

### Plant growth and competitive ability in EICA

The meta-analysis shows that plant vegetative production, but not fitness or competitive ability, is higher in the introduced range across invasive plant species as predicted by EICA. Many of the metrics of plant success reported in these studies as being greater in introduced-range than home-range populations have been shown to be greater in invasive species than in related native species (Grotkopp et al. 2002; McDowell 2002), or greater in invasive species than in native species from the invaded community (Pattison et al.^1998^; Smith and Knapp 2001). However, the relevance to plant invasions of higher growth of introduced plants in a common environment may be limited by the fact that experimental conditions rarely mimic natural plant communities. Plants grown under greenhouse conditions were typically grown alone in pots and experienced no competition, while the degree and form of competition in common gardens depended on garden design as well as resource availability (see Wilson and Tilman 1993) which was rarely reported or manipulated. For the purpose of this study, we assumed that growth under common conditions where competition was not manipulated was growth under noncompetitive conditions. There was no associated change in competitive ability across invasive species, which could be due to low sample size (Fig. 5), but is consistent with a study of 14 introduced species which found differences by ranges in plant biomass under noncompetitive, but not under low or highly competitive, conditions (Blumenthal and Hufbauer 2007). Overall, we found that introduced-range plants were more robust in terms of performance than home-range plants across invasive plant species as EICA predicts. However, our meta-analysis showed no general increase in fitness or competitive ability across the invasive plant species considered.

### Evolution in invasive species and recommendations for future research

There was broad support across these studies for evolutionary change in plant defense and performance occurring in concert with introduction and expansion in a new range, although not as predicted by EICA. Each of the six characteristics of plant defense or growth was significantly different between ranges when direction of trait change was disregarded, which suggests plant trait changes concurrent with range expansion should be considered as a component of invasion. Figure 4 shows that summary effect sizes in support of evolution are not only significant but larger in magnitude than the nonsignificant effect sizes generated by testing EICA. This indicates that invasive species evolve in terms of defense and performance traits without a general pattern toward greater or lower trait values across all invaders. Trait changes that confer success upon certain invasive plant species, for example, higher competitive ability, may not be crucial to the success of all invaders, for example, those plant species that establish populations in highly disturbed environments (Bossdorf et al. 2005).

The EICA research has focused primarily upon trait change within species, although the relative importance of stochastic forces as opposed to selective forces in this process (Keller and Taylor 2008) can provide a major focus to further research in the evolution of invasives. Stochastic events like founder's events can limit genetic variation, which was long assumed to limit the evolutionary potential of invasive species (Lee 2002). However, successive founding events across the landscape may also result in the loss of less successful genotypes and higher mean population and range trait values (Vasemagi 2006), and population bottlenecks that reduce variation may convert epistatic to additive variation for important phenotypic traits, increasing the rate of phenotypic change (Prentis et al. 2008). Accurate assessments of genetic variation for traits at high levels of organization such as ranges depend on accurate assessments of population-level and family-level variation, and the necessary nested analyses require common gardens with replication at the individual level as well as the population level (Conner and Hartl 2004). When common garden experiments are paired with molecular techniques, some evaluation of the relative importance of stochastic and selective forces is possible, using both the single-trait quantitative approaches (e.g., comparisons of F_ST_ and Q_ST_) and the quantitative genomic approaches (Beaumont and Balding 2004). Experimental crosses between home- and introduced-range plants may even allow quantitative trait loci (QTL) or genome mapping of traits correlated with invasiveness (Stinchcombe and Hoekstra 2007; Prentis et al. 2008). Such comparisons of home- and introduced-range plants are also important in addressing the influence of hybridization or changes in ploidy level, both of which are common in invasive populations, upon traits related to invasiveness (Prentis et al. 2008; Whitney and Gabler 2008). Previous studies comparing stochastic and selective influences on invasive evolution (Handley et al. 2008; van Kleunen and Fischer 2008) demonstrate that demographic and dispersal events cannot completely account for the evolutionary divergence of invasive plants from their ancestral home-range populations.

Selective factors including climate, resource availability, and biotic interactions may act in concert or in opposition upon ecologically important traits in invasive plant populations. In addition to quantitating the rate and strength of stochastic forces relative to selection, identifying the relative importance of environmental versus biotic selective agents should be a central topic of further research in evolution of invasive plants. Plant populations distributed across a wide geographic range may become locally adapted to climate factors correlated with latitude, both in native species (Macel et al. 2007; Kawakami et al. 2011) and in invasive species (Maron et al. 2004a,b). Including latitude as a covariate in models of plant performance has shown that for some invasive species, evolution of increased growth in invasive populations, which appeared to support EICA, was more closely correlated with latitude (Colautti et al. 2009). A recent study of the invasive flora of New South Wales used historical herbarium specimens to document that 70% of the 23 annual species accidentally introduced to the region have undergone significant changes in plant height, leaf shape, or specific leaf area over the last 150 years (Buswell et al. 2011). Interestingly, most of the plants that experienced a change in plant height since introduction were shorter than their introduced ancestors, which the authors attribute to selection by the arid climate in which reduced height means reduced water loss. Abiotic factors can act as strong selective forces, even in a relatively short-time period.

Recent research on feedbacks suggests that interactions between abiotic and biotic factors, including the environmental impacts of invasive plants, may also serve as selective forces for invasive species. Figure 4 shows that secondary chemistry varies by range, which in the framework of EICA suggests changes in herbivore pressure. However, models show that herbivory and resource availability may interact or act in opposition as selective forces on plant secondary metabolites (Zhang and Jiang 2006). Resource availability in the form of soil nutrients should be evaluated as a possible selective force for plant secondary chemistry, particularly as secondary metabolites can impact soil nutrient availability through effects on decomposition processes (Coley et al. 1985; Schweitzer et al. 2004). Altered nutrient cycling rates have been implicated as an ecosystem-level impact of invasive species (Ehrenfeld 2003), but should also be evaluated as an important evolutionary feedback for invasive plant species. Furthermore, while EICA only considers the biotic interactions of herbivores and plant competitors as selective forces, more recent research shows that soil biotic communities have the potential to act as selective agents, as certain tree species including invasive *Ailanthus altissima* cultivate soil biota beneficial to their offspring (Pregitzer et al. 2010; Felker-Quinn et al. 2011).

Further consideration of the possible evolutionary trajectories of plant invasions may inform how researchers determine the risk, impact, and management of invasive plant species. Evidence suggests that the enhancement of traits via evolution may be constrained by negative correlations between phenotypic traits that are subject to selection, for example in *Melaleuca quinquenervia*, in which of three leaf terpenoids under selection, cineole and viridiflorol are reduced in correlation with an increase in the terpenoid nerolidol in invasive range plants, despite predictions based on selection skewers analysis of increases in cineole and viridiflorol over time (Franks et al. 2012). If geographic variation in selection strength (Thompson 1997) combined with tradeoffs between traits under selection is common across invasive species, we may be seriously limited in our ability to predict the evolutionary potential of different species, which Whitney & Gabler (2008) identified as crucial to improving the invasive species predictive schemes (ISPS) developed to identify and exclude potential invaders in different regions. In some evolutionary scenarios, the destructive impact of invasive species upon native species may diminish over time, as has been documented for a chronosequence of the invasion of *Alliaria petiolata* (Lankau et al. 2009), or a comparison between the impact of home- and introduced-range *Centaurea maculosa* on native grass species (Callaway et al. 2005). Studies that attempt to address the evolution of invasiveness should be careful to identify heritable traits associated with plant success and invasive impacts, to elucidate the mechanisms of selection that operate upon invasives, and to place selection firmly within the context of other evolutionary forces. The extent to which these goals are pursued will determine the extent to which our understanding of the evolution of invasive plant species informs management and prevention of the ecological impact of invasive plant species.
